# Use of bandage contact lenses in dogs with spontaneous chronic corneal epithelial defect after interventional treatment

**DOI:** 10.18849/ve.v11i1.731

**Published:** 2026-01-26

**Authors:** Charlie Batchelor+

**Keywords:** CANINE, CORNEAL ULCERS, CONTACT LENS, EYE, OPHTHALMOLOGY, SPONTANEOUS CHRONIC CORNEAL EPITHELIAL DEFECT

## Abstract

**PICO question:**

In dogs with spontaneous chronic corneal epithelial defects (SCCEDs), does the use of a bandage contact lens (BCL) after interventional treatment compared to treatment alone decrease the time to clinical resolution?

**Clinical bottom line:**

**Category of research:**

Treatment.

**Number and type of study designs reviewed:**

Three studies were reviewed, one of which was a prospective non-randomised observational study, one was a case control study, and one was a prospective randomised controlled trial.

**Strength of evidence:**

Strong.

**Outcomes reported:**

All studies demonstrated appropriate healing, with three studies showing a shorter corneal healing time with the use of a bandage contact lens. No studies demonstrated a longer or similar healing time with use of a bandage contact lens.

**Conclusion:**

In cases of canine SCCEDs, application of a bandage contact lens after interventional treatment shortens corneal healing time.

How to apply this evidence in practice

The application of evidence into practice should take into account multiple factors, not limited to: individual clinical expertise, patient’s circumstances and owners’ values, country, location or clinic where you work, the individual case in front of you, the availability of therapies and resources.

Knowledge Summaries are a resource to help reinforce or inform decision making. They do not override the responsibility or judgement of the practitioner to do what is best for the animal in their care.

## Clinical scenario

You are presented with a 5-year-old, male neutered Boxer dog with corneal oedema and blepharospasm. On ocular examination, you identify a spontaneous chronic corneal epithelial defect (SCCED), which you plan to treat under local anaesthetic using a cotton bud to remove loose epithelial edges followed by a diamond burr debridement or other interventional therapy. With the possibility that this may take weeks or months to heal, you wonder whether there is something else you can do to reduce the time this takes to resolve.

## The evidence

Historically, bandage contact lenses (BCL) have been used in canine patients with the hypothesis of providing a ‘scaffold’ for corneal regeneration and healing while providing environmental protection (Heinrich, 2014). However, no control or non-intervention groups were used in studies prior to 2015 in the literature search. Studies from 2015 onwards filled this knowledge gap to provide the clinical evidence for what was not previously supported by good quality, methodologically robust evidence.

Three studies directly relevant to the PICO question were reviewed (Dees et al., 2017; Rivera-Viscal et al., 2024; Wooff & Norman, 2015) to assess their contribution towards answering the question posed.

The strength of the combined evidence provided by the three studies is strong, with all studies demonstrating a shorter healing time for SCCEDs when treated using a BCL. All three studies have different study designs with two prospective studies (one randomised (Wooff & Norman, 2015) and one non-randomised (Dees et al., 2017)) and one case control study (Rivera-Viscal et al., 2024). The two prospective studies bring forth a stronger evidence base for changes to clinical practice, however the prospective, randomised study (Wooff & Norman, 2015) has a much smaller sample size which does restrict its broader applicability.

## Summary of the evidence

## Table 1

**Dees et al. (2017):** Effect of bandage contact lens wear and postoperative medical therapies on corneal healing rate after diamond burr debridement in dogs **Aim: **To determine the effect of bandage contact lens wear and type of postoperative medical treatment on corneal healing rates in dogs after diamond burr debridement.

Population:	Canine patients that had undergone diamond burr debridement (DBD) for treatment of spontaneous chronic corneal epithelial defects (SCCEDs).
Sample size:	237 dogs, all dogs contributed one eye to the study.
Intervention details:	Dogs were split into 12 groups depending on a single postoperative antibiotic (ofloxacin (89/237), tobramycin (79/237) or oxytetracycline/polymyxin B (69/237)) in combination with sodium chloride ointment and/or a bandage contact lens (BCL). 6 out of the 12 groups used a bandage contact lens (129/237). The lens was based on best fit after measuring (Acrivet D2/D4/D9 or Bausch and Lomb Plano T lens with 8.6 mm curve). All dogs had diamond burr debridement to remove non-adherent loose epithelium. Patients were examined (approximately) every 7 days.
Study design:	Prospective, non-randomised observational study.
Outcome Studied:	Patients were examined under ophthalmic fluorescein examination with success being defined as non-retention of fluorescein – objective assessment.
Main Findings (relevant to PICO question):	When comparing all combinations of antibiotic, sodium chloride and use of a bandage contact lens, contact lens use and retention significantly improved healing times for every group regardless of the other experimental protocol used. The median healing time with use of BCL was approximately three days shorter when compared to groups without BCL (P = 0.0002). Patients were examined under ophthalmic fluorescein examination with success being defined as non-retention of fluorescein – objective assessment.
Limitations:	Post intervention checks were at approximate, rather than strict intervals due to owner scheduling. Time to healing was measured in bounds of 7 days rather than exact day to healing. Patients were not examined daily. Some dogs required a second procedure (41/237). Lens retention rate was 62% – different makes and sizes were used to accommodate differences between individuals. One brand was retained better than the other, possibly due to better initial fitting. Three dogs with a concurrent endocrinopathy (diabetes) were included in the study, along with five cases of infectious keratitis and one case of keratoconjunctivitis sicca – all of which would have had changes in healing rate when compared to baseline but were included in statistical analysis. Patient groups were not randomised – placed into treatment groups based on medication and BCL availability at time of treatment. Owners and ophthalmologists were not blinded to the intervention group. No exclusion criteria are discussed in the paper. The Bausch and Lomb lenses were retained significantly more frequently than the Acrivet lenses. (P < 0.0001).

## Table 2

**Rivera-Viscal et al. (2024):** Effect of owner presence on healing of spontaneous chronic corneal epithelial defects after anterior stromal puncture **Aim: **To examine the effect of client presence on healing rates of spontaneous chronic corneal epithelial defect (SCCEDs) following debridement and anterior stromal puncture (ASP).

Population:	Client owned dogs presented with at least a 7-day history of a fluorescein retaining spontaneous chronic corneal epithelial defect (SCCED) with non-adherent epithelium. All took place at a single location (University of Wisconsin Veterinary Care).
Sample size:	68 dogs.
Intervention details:	SCCED was diagnosed at first appointment, non-adherent epithelium was removed with a cotton tipped applicator and then an anterior stromal puncture procedure was performed. A bandage contact lens was placed (Bausch and Lomb Plano T lens with 8.6 mm curve). 33 bandage contact lenses (BCLs) were placed. All patients received topical antibiotic therapy, systemic pain management and an Elizabethan collar.
Study design:	Case control study – although primarily looking at investigating the impact of the presence or absence of the client in the room at time of intervention.
Outcome Studied:	Patients were examined under ophthalmic fluorescein examination with success being defined as non-retention of fluorescein – objective assessment.
Main Findings (relevant to PICO question):	BCL placement (33/68) was associated with significantly greater odds of corneal healing by first recheck timepoint (median 14 days) (OR = 4.00; 95% CI: 1.43–11.2; P = 0.008).
Limitations:	Not all dogs were placed on the same antibiotic regimen. Not all records contained information as to whether the BCL was present at time of first recheck. Each procedure was not always performed by the same clinician. Paper discusses more BCLs placed in the non-client group, perhaps because clinicians were more comfortable placing lenses with clients not in the room. This could affect the overarching intention of this study. Exclusion criteria were concurrent ocular conditions and/or endocrinopathies.

## Table 3

**Wooff & Norman (2015):** Effect of corneal contact lens wear on healing time and comfort post LGK for treatment of SCCEDs in boxers **Aim: **To determine whether dogs with spontaneous chronic corneal epithelial defects (SCCEDs) would heal faster and with an improved comfort score following linear grid keratotomy (LGK) combined with corneal contact lens wear when compared to dogs having the LKG procedure alone.

Population:	Boxer dogs presented to a single referral ophthalmology centre and diagnosed with a SCCED.
Sample size:	27 dogs, 27 eyes.
Intervention details:	In all eyes, a linear grid keratotomy was performed under sedation after the loose epithelial edges were removed using a scalpel or Kimura spatula. 14 eyes were randomly assigned to receive BCL (Acrivet size D2/D3). A temporary lateral tarsorrhaphy was performed in all cases to minimise corneal exposure.
Study design:	Prospective, randomised controlled trial (group assigned by computer software).
Outcome Studied:	Patients were examined under ophthalmic fluorescein examination with success being defined as non-retention of fluorescein. Surveys were completed by owners to assess comfort level and contact lens retention.
Main Findings (relevant to PICO question):	Eyes with BCLs (14/27) had a significant (P* = *0.035) decrease in median corneal healing time (7 days) when compared to those without (10 days). No difference in comfort scores between groups.
Limitations:	Multiple observers for comfort scoring. Tarsorrhaphy could have confounded scores for blepharospasm when considering comfort. Small study population. Information on power calculation should have been included. Inconsistent follow up with some dogs due to scheduling conflicts. Some patients on systemic NSAIDs (previously prescribed). Not all antimicrobial therapy was the same. All patients continued on their previously prescribed topical antibiotic. Dogs with comorbidities that would influence corneal healing were excluded from the study.

## Appraisal, application and reflection

Corneal ulcerative disease is a common presentation in first opinion practice for both canine and feline patients (O’Neill et al. (2017). With reflective practice being an important part of professional development, it is prudent to identify where changes and additions to protocols can be made to improve patient outcomes over time and drive quality improvement.

O’Neill et al. (2017) demonstrates that over a single year, when compared to crossbreed dogs, brachycephalic dogs have 11.18 times the odds and spaniels have 3.13 times the odds of corneal ulcerative disease. As brachycephalic dogs and spaniel breeds remain popular, corneal disease and exposure keratopathies are a continuing concern for our small animal patients. O’Neill et al. (2017) states the overall incidence of corneal disease as 0.8% of consultations, with 7.4% of these being referred onward for advanced management strategies (not explicitly defined in this study). This demonstrates that the majority of cases of corneal ulcerative disease are managed solely in primary care practice.

The first instance of contact lenses being used in the management of corneal disease in the literature is Schmidt et al. (1977). Whilst it is a reasonable preliminary study that provides information about the safety of bandage contact lenses in canine patients, there were limitations to the study due to the lack of a control group or a solid experimental plan. Case studies start to appear in the literature through the 1980s and 1990s (for example Wolfer & Grahn, 1994), with use being described in textbooks and review papers from 1990 onwards (Kirschner, 1990; Heinrich, 2014).

With regard to the studies identified by the search process, there was a wide variety of quality demonstrated and it was interesting to note the changes in process over time from the non-comparative experimental study of Schmidt et al. (1977) through to the randomised controlled trial of Wooff & Norman (2015). Some of the more recent studies showed more appropriate statistical analysis, along with standardised sample groups and control groups.

The Rivera-Viscal et al. (2024) study contains evidence which is relevant to answering the PICO question; however the initial objective of that study was to focus on the effect of client presence/absence at time of intervention on corneal healing rates of SCCEDs. The absence of a client at the time of intervention was associated with faster healing – however, more of the patients where the client was absent had a bandage contact lens placed. This is discussed in the publication itself as a likely confounder. Regardless, the data analysis supports the PICO of this Knowledge Summary: that bandage contact lens application is associated with quicker healing of SCCEDs.

It is also of note that despite three different interventions being brought forth by the database search – anterior stromal puncture (Dees et al*.*, 2017), diamond burr debridement (Rivera-Viscal et al., 2024) and linear grid keratotomy (Wooff & Norman, 2015) – all three papers demonstrate that use of a BCL leads to a faster clinical resolution.

An important observation, as discussed in Grinninger et al. (2015), is that not all eyes need the same size of contact lens. However, all sizes of canine patients in the study (from a Maltese to a Rottweiler) were able to be accommodated with just four sizes of contact lens. If this finding is matched in further studies, perhaps BCLs could be considered a reasonable adjunctive treatment in first opinion medicine rather than being the preserve of referral ophthalmology practice. Another benefit of this would be a smaller financial outlay for stocking BCLs in practice, with more patients potentially benefitting from their use in management of their SCCED.

Unfortunately, the retention rate of BCLs can vary and is a notable issue with dogs of a brachycephalic or exophthalmic conformation when compared to mesocephalic dogs (Grinninger et al., 2015), but these are the majority of patients presented for ocular issues in primary care practice (O’Neill et al., 2017). For these patients, BCL placement can be incorporated into the first opinion setting, but it is important to note that placing, sizing and removing a BCL requires a degree of training. A corneal cytology should always be performed to identify any cytological evidence of infection. This is important as BCLs should not be placed in cases of bacterial keratitis due to the potential risk of keratomalacia, nor should they be used in cases of keratoconjunctivitis sicca (Pratumjorn et al., 2022).

## Methodology

**Search Strategy:** 

Databases searched and dates covered:	CAB Abstracts on OVID Platform (1973 to 2025 Week 5) PubMed via the NCBI website (1990 to January 2025)
Search strategy:	CAB Abstracts:“Contact lens” AND (ulcer* OR “corneal epithelial defect”) AND (dog OR canine) PubMed:(("contact lens") AND (ulcer* OR "corneal epithelial defect")) AND (dog OR canine)
Dates searches performed:	27 January 2025

**Exclusion / Inclusion Criteria:** 

Exclusion:	Articles focusing on other interventions, articles not using contact lenses, articles in a language other than English, articles not available digitally, articles regarding other species, articles discussing a single case outcome, articles with no interventional comparator.
Inclusion:	Articles that were relevant to the PICO.

**Search Outcome:** 

Database	Number of results	Excluded – different intervention	Excluded – language other than English	Excluded – full text not available	Excluded – single case study	Excluded – no comparator	Excluded – not relevant	Total relevant papers
CAB Abstracts	14	4	1	2	3	2	0	2
PubMed	11	2	0	0	2	2	2	3
Total relevant papers when duplicates removed								3

## ORCiD

Charlie Batchelor: https://orcid.org/0009-0005-5871-8538

## Conflict of Interest

The author declares no conflicts of interest.

## Contribute to the evidence

There are two main ways you can contribute to the evidence base while you are enhancing your CPD:

- Tell us your information need
- Write a Knowledge Summary

Either way, you will be helping to add to the evidence base, and strengthen the decisions that veterinary professionals around the world make to give animals the best possible care. Learn more here: https://veterinaryevidence.org/index.php/ve/author-hub
